# Angiotensin receptor blockade and stereotactic body radiation therapy for early stage lung cancer ARB & SBRT for early stage lung cancer

**DOI:** 10.1080/15384047.2022.2126250

**Published:** 2022-10-06

**Authors:** Lauren T. Maloney, Emile Latour, Yiyi Chen, Douglas Rice, Alison Grossblatt-Wait, Nima Nabavizadeh, Charles R. Thomas, Kristina H. Young, Joshua M. Walker, John Holland, Aaron J. Grossberg

**Affiliations:** a School of Medicine, Oregon Health & Science University, Portland, OR, USA; b Biostatistics Shared Resource, Oregon Health and Science University, Portland, OR, USA; c Brenden Colson Center for Pancreatic Care, Oregon Health & Science University, Portland, OR, USA; d Cancer Early Detection Advanced Research Center, Knight Cancer Institute, Oregon Health & Science University, Portland, OR, USA; e Department of Radiation Medicine, Oregon Health & Science University, Portland, OR, USA; f Department of Radiation Oncology, Dartmouth Hitchcock Medical Center, Lebanon, NH, USA; g Earle A. Chiles Research Institute, Providence Cancer Institute, Portland, OR, USA; h The Oregon Clinic, Radiation Oncology Division, Portland, OR, USA; i Department of Cell, Developmental & Cancer Biology, Oregon Health & Science University, Portland, OR, USA

**Keywords:** Non-small cell lung cancer (NSCLC), stereotactic body radiation therapy (SBRT), angiotensin receptor blocker (ARB), tumor growth factor beta (TGF-β)

## Abstract

Stereotactic body radiotherapy (SBRT) demonstrates excellent local control in early stage lung cancer, however a quarter of patients develop recurrence or distant metastasis. Transforming growth factor-beta (TGF-β) supports metastasis and treatment resistance, and angiotensin receptor blockade (ARB) indirectly suppresses TGF-β signaling. This study investigates whether patients taking ARBs while undergoing SBRT for early stage lung cancer exhibited improved overall survival (OS) or recurrence free survival (RFS) compared to patients not taking ARBs. This was a single institution retrospective analysis of 272 patients treated with SBRT for early stage lung cancer between 2009 and 2018. Patient health data was abstracted from the electronic medical record. OS and RFS were assessed using Kaplan–Meier method. Log-rank test was used to compare unadjusted survival between groups. Univariable and multivariable Cox proportional hazard regression models were used to estimate hazard ratios (HRs). Of 247 patients analyzed, 24 (10%) patients took ARBs for the duration of radiotherapy. There was no difference in mean age, median tumor diameter, or median biologic effective dose between patients taking ARBs or not. Patients taking ARBs exhibited increased OS (ARB = 96.7 mo.; no ARB = 43.3 mo.; HR = 0.25 [95% CI: 0.10 to 0.62, *P* = .003]) and increased RFS (median RFS, ARB = 64.3 mo.; No ARB = 35.1 mo.; HR = 0.26 [95% CI: 0.10 to 0.63, *P* = .003]). These effects were not seen in patients taking angiotensin converting enzyme inhibitors (ACEIs) or statins. ARB use while undergoing SBRT for early stage lung cancer may increase OS and RFS, but ACEI use does not show the same effect.

## Introduction

For patients with early stage non-small cell lung cancer (NSCLC), stereotactic body radiotherapy (SBRT) provides excellent local control and acceptable toxicity, leading to its increasing use as primary therapy in both operable and medically inoperable patients.^1,2^ Despite this, about 40% of patients develop recurrent disease, with approximately half of recurrences occurring distantly.^3^ Therefore, many groups are investigating factors that drive metastasis from early stage NSCLC tumors, in the hopes of identifying therapeutic approaches to reduce distant recurrence risk.

Transforming growth factor-beta (TGF-β) is a cytokine that enhances tumor progression by mediating epithelial to mesenchymal transition (EMT),^4^ tumor cell motility and metastasis,^5^ and immunosuppression.^6–8^ TGF-β is generated by both cancer cells and the microenvironment, and its release is amplified in response to radiation treatment.^9^ TGF-β suppresses CXCR3 expression on CD8 + T cells, thereby preventing effective T cell trafficking to tumors.^10^ TGF-β inhibition enhances radiation sensitivity in multiple preclinical tumor models by attenuating the DNA damage response and reversing immunosuppression.^11^ Additionally, blocking TGFb may lead to improved outcomes by slowing or preventing EMT. Thus, TGF-β inhibition is hypothesized to improve local and distant cancer control by acting on tumor cells, the microenvironment, and the anti-tumor immune response. As such, incorporation of anti-TGF-β therapies is being evaluated in ongoing clinical trials in several solid tumors, with some promising results in early phase clinical studies.^12^

Angiotensin II receptor blockade (ARB) medications are a commonly prescribed antihypertensive medication class with a well-established safety profile and known anti-TGF-β effects.^13^ ARBs indirectly suppress TGF-β signaling by blocking thrombospondin-1 mediated activation of latent TGF-β.^14^ Patients with advanced NSCLC undergoing platinum based chemotherapy had improved median survival when concurrently taking ARBs or angiotensin converting enzyme inhibitors (ACEIs).^15^ Furthermore, inclusion of ARBs in neoadjuvant treatment demonstrated encouraging results in a prospective phase II single arm study in patients with locally advanced pancreatic cancer.^16^ Investigating the effects of ARBs on outcomes of patients with early stage NSCLC treated with SBRT offers a real-world opportunity to evaluate the potential efficacy of TGF-β blockade on long-term cancer control in a yet unevaluated patient population. Based on the known effects of ARBs to reduce TGF-β release, we hypothesized that patients undergoing SBRT for early stage NSCLC have increased survival and decreased recurrence compared to patients not on ARBs. Secondarily, given the role that TGF-β plays in mediating radiation-induced pulmonary fibrosis,^17,18^ we investigated associations between ARB use and the development of pulmonary fibrosis and pneumonitis following SBRT.

## Methods

### Patient cohort

This study included patients with presumed or biopsy proven early stage NSCLC treated with SBRT at a single academic hospital from January 2009–December 2018. All patients were treated to a BED > 100 Gy in 3–8 fractions. Patients with a diagnosis of small cell lung cancer, pulmonary metastases treated with SBRT, or Stage III–IV lung cancer were excluded. A total of 247 consecutive patients were identified and included in the analysis. Clinical data were abstracted from the electronic medical record. We reviewed medication records before, during, and after radiotherapy treatment and included ARBs, ACEIs and statins to account for non-cancer related impacts of these medications on survival. All patients included in these treatment groups were actively taking the indicated drug prior to, during, and for at least 6 months following the completion of SBRT.

CT simulation was performed on a Brilliance CT Big Bore (Philips Medical Systems, Amsterdam, Netherlands) with patient in a supine position, immobilized with either the BodyFIX (Elekta, Atlanta, GA) or Vac-Lok (Civco, Coralville, IA) vacuum cushion systems. Patients were imaged from the cricoid to below the diaphragm using a 1–3 mm slice thickness, and motion management was performed using deep inspiratory breath hold (DIBH) or four-dimesional CT (4DCT). Planning target volume (PTV) consisted of a symmetric 5 mm margin on gross tumor volume in cases where DIBH was utilized or the same margin on an internal target volume encompassing the maximum tumor volume in all dimensions in cases where 4DCT was utilized. Both static field intensity-modulated radiotherapy and volumetric modulated arc therapy were utilized and cases were planned to ensure 95% of the PTV was covered by the prescription dose.

Overall survival (OS) is defined as the primary outcome in this study, and calculated as the time from treatment start until death from any cause. Recurrence free survival (RFS) is defined as the secondary outcome, and calculated as time from treatment start until the first of recurrence or death from any cause. Toxicity outcomes for patients, including pulmonary fibrosis and radiation pneumonitis, were determined using the Common Terminology Criteria for Adverse Events version 4.0. Toxicity outcomes were documented while patients were on treatment and in a six month window post last radiation treatment. Charlson Comorbidity Index was calculated for each patient using the R-package ‘comorbidity’^19^ by utilizing ICD 10 codes extracted from medical records, based on the methodology described by Deyo and colleagues.^20^

## Statistical analysis

Differences between groups were assessed using two-sample *t-*tests (for continuous variables) and Fisher’s Exact tests (for categorical variables). Continuous data are presented as mean ± standard deviation (SD) or median [interquartile range (IQR)]; and categorical data as counts and percentages. We used the Kaplan–Meier method to estimate survival curves and performed log-rank test to compare unadjusted survival distributions between groups of patients. Univariable and multivariable analyses for OS and RFS were conducted using Cox proportional hazards models, on which the hazard ratios (HRs) were estimated. Variables were screened for inclusion in the multivariable model if univariable Cox regression analysis revealed *P* < .25. Data were graphed and analyzed using the software package R: A Language and Environment for Statistical Computing. *P*-values < .05 were regarded as statistically significant.

**Figure 1.**
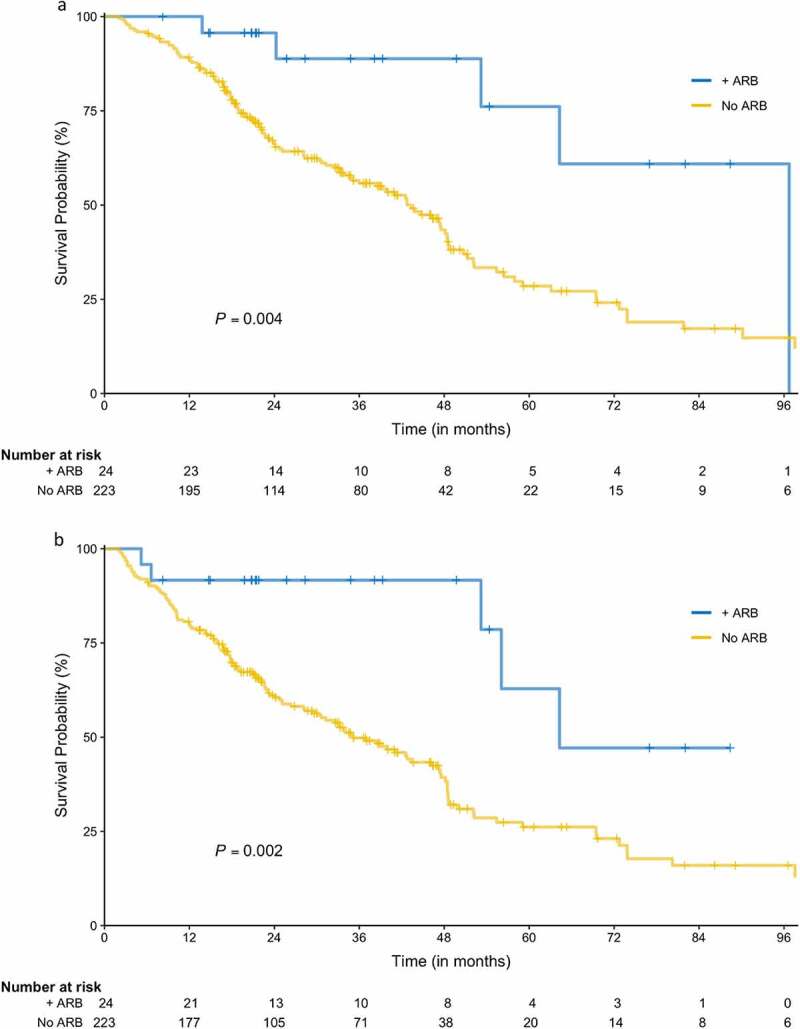
Overall and recurrence-free survival. (a) Overall survival (+ARB arm is denoted by the blue line, No ARB by the gold line). (b) Recurrence-free survival (+ARB arm is denoted by the blue line, No ARB by the gold line).

### Results

## Patient characteristics

A total of 247 patients were included in the analysis. Of these patients, 31 (12.6%) were female and 216 (87.4%) were male. The average age was 73.5 ± 8.4 years. Nine patients (3.7%) were never smokers, 128 (52.2%) were former smokers, and 108 (44.1%) were current smokers. 24 patients (9.7%) were taking an ARB medication during their treatment, whereas 67 patients (27.1%) were on ACEIs, and 113 patients (45.7%) were on statins. 195 (79%) patients had T1 tumors, and 52 (21%) had T2 or T3 disease (Table 1). Charlson Comorbidity Index was not different in patients on ARBs than patients who were not on ARB medications (*P* = .535). There were no significant differences in other baseline patient characteristics or clinical variables between patients treated with ARBs and those not treated with ARBs.

**Table 1.** Patient characteristics.

		ARB during			
Characteristic	Number available	All subjects	Yes	No	*P*-value
n		247	24	223	
Age, years (mean (SD))	247	73.5 (8.4)	74.0 (7.2)	73.4 (8.5)	0.729
BED (median [IQR])	247	115.5 [100.0, 132.0]	110.3 [103.7, 132.0]	115.50 [100.00, 132.00]	0.304
Sex (%)	247				0.199
Female		31 (12.6)	5 (20.8)	26 (11.7)	
Male		216 (87.4)	19 (79.2)	197 (88.3)	
Smoking Hx (%)	245				0.678
Never		9 (3.7)	1 (4.2)	8 (3.6)	
Former		128 (52.2)	14 (58.3)	114 (51.6)	
Current		108 (44.1)	9 (37.5)	99 (44.8)	
Histology (%)	247				0.796
Presumed		158 (64.0)	14 (58.3)	144 (64.6)	
SCC		36 (14.6)	4 (16.7)	32 (14.3)	
Adeno		52 (21.1)	6 (25.0)	46 (20.6)	
BAC		1 (0.4)	0 (0.0)	1 (0.4)	
NOS		0 (0.0)	0 (0.0)	0 (0.0)	
Deceased? (%)	247				0.002
Yes		129 (52.2)	5 (20.8)	124 (55.6)	
No		118 (47.8)	19 (79.2)	99 (44.4)	
Recurrence? (%)	247				0.305
Yes		56 (22.7)	3 (12.5)	53 (23.8)	
No		191 (77.3)	21 (87.5)	170 (76.2)	
Recurrence location (%)	53				0.060
Local		6 (11.3)	0 (0.0)	6 (12.0)	
Regional		17 (32.1)	3 (100.0)	14 (28.0)	
Distant		30 (56.6)	0 (0.0)	30 (60.0)	
ACEI during (%)	247				0.007
Yes		67 (27.1)	1 (4.2)	66 (29.6)	
No		180 (72.9)	23 (95.8)	157 (70.4)	
Statin during (%)	113				1.000
Yes		113 (100.0)	16 (100.0)	97 (100.0)	
No		0 (0.0)	0 (0.0)	0 (0.0)	
Tumor size (cm) (median [IQR])	247	2.10 [1.60, 2.80]	2.20 [1.62, 3.25]	2.10 [1.60, 2.80]	0.656
Tumor stage (%)	247				0.184
T1		195 (78.9)	16 (66.7)	179 (80.3)	
T2-3		52 (21.1)	8 (33.3)	44 (19.7)	
Charlson Comorbidity Index (median [IQR])	243	2.00 [1.00, 3.00]	1.00 [1.00, 3.00]	2.00 [1.00, 3.00]	0.535

Clinical and demographic characteristics of patients. *Abbreviations*: BED, biologically effective dose; ACEI, angiotensin converting enzyme inhibitor; SCC, Squamous cell carcinoma; Adeno, Adenocarcinoma; BAC, Bronchioalveolar carcinoma; ARB, Angiotensin Receptor Blocker.

## Overall survival

The number of total deaths among all patients was 129 (52.2%); 5 (20.8%) in the ARB group and 124 (55.5%) in the no ARB group. Median follow up time for OS was 43.6 months [95% CI: 38.6 to 48.5]; 34.8 months [95% CI: 21.3 to 54.4] in the ARB group and 45.9 months [95% CI: 38.9 to 48.9] in the no ARB group (*P* = .204). The ARB group exhibited significantly improved OS compared with the no ARB group (median 96.7 months [95% CI: 53.2 to not estimable] vs 43.3 months [95% CI: 34.8 to 48.4]; log-rank test *P* = .004; Figure 1A). On univariable analysis, ARB use, tumor size, and tumor histology were associated with OS, whereas neither ACEI nor statin use was associated with OS (Supplementary Table 1). On multivariable analysis, only ARB use and tumor size were associated with OS, and the adjusted HR for the ARB group vs no ARB was 0.25 [95% CI: 0.10 to 0.62] (*P* = .003; Table 2).

**Table 2.** Overall survival multivariable analysis.

	Unadjusted		Adjusted	
Covariate	HR (95% CI)	*P*-value	HR (95% CI)	*P*-value
ARB during		0.001		< 0.001
No	Reference level		Reference level	
Yes	0.29 (0.12 to 0.72)	0.007	0.25 (0.10 to 0.62)	0.003
Histology		0.005		0.051
Presumed	Reference level		Reference level	
SCC	2.37 (1.48 to 3.79)	< 0.001	1.87 (1.11 to 3.15)	0.018
Adeno	1.38 (0.91 to 2.10)	0.130	1.51 (0.99 to 2.31)	0.054
BAC	4.32 (0.59 to 31.41)	0.148	3.49 (0.48 to 25.47)	0.218
Tumor size (cm)		< 0.001		0.012
	1.37 (1.15 to 1.63)		1.30 (1.06 to 1.58)	

Overall Survival Survival Multivariable Analysis. *Abbreviations*: SCC, Squamous cell carcinoma; Adeno, Adenocarcinoma; BAC, Bronchioalveolar carcinoma; HR, Hazard Ratio; ARB, Angiotensin Receptor Blocker.

## Recurrence-free survival

The number of total recurrences among all patients was 56 (22.7%); 3 (12.5%) in the ARB group and 53 (23.8%) in the no ARB group. Over half of the recurrences (30, 56.6%) occurred in distant sites, with 17 (32.1%) regional recurrences and 6 (11.3%) local recurrences. Patients taking ARBs had no local or distant recurrences. Median follow up time for RFS was 41.4 months [95% CI: 38.1 to 48.9], 31.6 months [95% CI: 20.8 to 54.4] in the ARB group and 45.9 months [95% CI: 38.6 to 49.3] in the no ARB group (*P* = .106). RFS was significantly longer in patients taking ARBs compared with the no ARB group (median 64.3 months [95% CI: 53.2 to not estimable] vs 35.1 months [95% CI: 28.3 to 46.2]; log-rank test *P* = .002; Figure 1B). On univariable analysis, ARB use, tumor size, tumor histology and tumor stage were associated with RFS (Supplementary Table 2). Again, niether ACEI nor statin use was associated with RFS. On multivariable analysis, only ARB use and histology were associated with RFS. The adjusted HR for the ARB group was 0.26 [95% CI: 0.10 to 0.63] compared to the no ARB group (*P* = .003; Table 3).

**Table 3.** Recurrence free survival multivariable model.

	Unadjusted		Adjusted	
Covariate	HR (95% CI)	*P*-value	HR (95% CI)	*P*-value
ARB during		< 0.001		< 0.001
No	Reference level		Reference level	
Yes	0.27 (0.11 to 0.67)	0.005	0.26 (0.10 to 0.63)	0.003
Histology		0.009		0.004
Presumed	Reference level		Reference level	
SCC	2.17 (1.37 to 3.42)	< 0.001	2.19 (1.39 to 3.46)	< 0.001
Adeno	1.44 (0.97 to 2.16)	0.072	1.60 (1.07 to 2.40)	0.022
BAC	3.39 (0.47 to 24.53)	0.227	3.19 (0.44 to 23.07)	0.251

Recurrence Free Survival Multivariable Analysis. *Abbreviations*: SCC, Squamous cell carcinoma; Adeno, Adenocarcinoma; BAC, Bronchioalveolar carcinoma; HR, Hazard Ratio; ARB, Angiotensin Receptor Blocker.

## Toxicity

Pneumonitis developed in 33 patients (13.4%), of whom 29 (11.7%) had grade 1 and 4 (1.6%) had grade 2 fibrosis. We observed no difference in rate or severity of pneumonitis associated with ARB use (*P* = .456, Table 3). Pulmonary fibrosis was documented in 40 patients (16.2%), with 34 patients (13.8%) developing grade 1 and 6 (2.4%) developing grade 2 fibrosis. Neither the rate nor severity of pulmonary fibrosis was different between the ARB and no ARB groups (*P* = .651; Table 4).

**Table 4.** Radiation-induced late toxicities.

Characteristic	Number available	Arb during			*P*-value
		All subjects	Yes	No	
Pulmonary fibrosis	247				0.651
0		207 (83.8)	20 (83.3)	187 (83.9)	
1		34 (13.8).	3 (12.5)	31 (13.9)	
2		6	1 (4.2)	5 (2.2)	
Pneumonitis grade	247				0.456
0		214 (86.6)	21(87.5)	193 (86.5)	
1		29 (11.7)	2 (8.3)	27 (12.1)	
2		4 (1.6)	1 (4.2)	3 (1.3)	

Abbreviations: ARB, Angiotensin Receptor Blocker

### Discussion

We found that patients who took ARBs in conjunction with SBRT for early stage NSCLC exhibited a doubling in OS and RFS compared to patients who did not take ARBs. No patients on ARBs during treatment developed local or distant recurrence during the follow up period of this study, suggesting that ARB use may improve systemic tumor control. Notably, these effects were not seen with the other antihypertensive or lipid-lowering agents. This work builds on previous findings which show improved survival in late stage NSCLC patients taking ACEi or ARBs while undergoing platinum based chemotherapy.^15^

A 2019 retrospective study found that patients who had undergone SBRT for early stage NSCLC were at increased risk for major cardiac events, including cardiac death, unstable angina, and myocardial infarction.^21^ This work concluded that cardiac radiation dose is an independent predictor of cardiac events, building on an existing body of literature establishing cardiac events as a major cause of death in lung cancer patients.^21–23^ ACEIs, ARBs and statins are all classes of drugs which reduce mortality from cardiovascular causes, yet only ARB use is associated with improved OS and RFS.^13^ There was no difference in age or comorbidities, assessed by Charlson Comorbidity Index, among groups, indicating that the competing risks of death were similar between the ARB and no ARB groups. Furthermore, no local or distant recurrences were observed in patients taking ARBs. The few regional recurrences recorded in the ARB group likely represent untreated occult micrometastatic nodal disease, as SBRT does not include regional fields and no patients received systemic therapy. Together, this suggests that the reduced mortality in this patient population is most likely due to a cancer-specific treatment effect of ARBs and not a reduction in competing causes of death.

The main pro-tumorigenic effects of angiotensin II, including angiogenesis, vascular remodeling, proliferation and inflammation, are mediated through the type 1 angiotensin receptor (AT_1_R).^24^ Binding of angiotensin II to AT_1_R results in increased transcription of *TGFB1* mRNA and TGF-β cleavage from binding proteins in the extracellular matrix.^24–26^ Therefore, blockade of angiotensin II signaling through AT_1_R would be expected to decrease TGF-β levels, leading to increased radiosensitivity and decreased metastatic potential. To this end, there are 17 open studies in the United States listed on clinicaltrials.gov utilizing TGF-β blockade and other therapies to treat NSCLC as of the writing of this manuscript. Although we did not have banked specimens to measure TGF-β in our patient population, our data provide preliminary real world support for the combinatorial use of these agents in prospective early stage NSCLC studies. Furthermore, blocking TGF-β may lead to improved outcomes by slowing or preventing EMT. As previous research in this area has focused on patients undergoing palliative platinum based chemotherapy in late stage NSCLC, this work suggests a novel intervention to target a patient population undergoing SBRT at a much earlier disease stage.^15^ Both platinum based chemotherapy and radiation rely on DNA damage to exert anti-tumor effect, suggesting a possible shared mechanism between the improved survival with ARBs in this report and Wilop and colleagues’ retrospective study.^15^ Importantly, although ARBs impact TGF-β signaling, we were unable to confirm that the association between treatment and outcome was mediated by TGF-β antagonism in this study, so other pathways may drive this association.

The specificity of the improved outcomes to ARBs suggests that differences in the mechanisms of action between ARBs and ACEIs could provide insight into the mediators of this effect. Both ACEIs and ARBs derive their antihypertensive effects by decreasing the vasoconstrictive activity of angiotensin II, which is mediated by AT_1_R. Whereas ARBs function as specific inhibitors of the AT_1_R, ACEIs block the conversion of angiotensin I to angiotensin II, thereby preventing signaling through both the AT_1_R and type 2 angiotensin receptor (AT_2_R). Although less is known about the downstream effects of AT_2_R, activation of this receptor is thought to counterbalance the effects of AT_1_R, with ligand binding inducing an anti-inflammatory, anti-fibrotic, anti-proliferative response.^27^ In preclinical lung cancer models, agonists at AT_2_R or nanoparticle-based AT_2_R gene therapy attenuated tumor growth.^27,28^ We therefore speculate that unopposed signaling through the AT_2_R in the context of ARB treatment provides a second potential mechanistic explanation for the improved outcomes associated with ARBs, but not ACEIs. Notably, a recent meta-analysis revealed an increase in the risk for developing lung cancer associated with long-term ARB use, thought to be mediated by carcinogenic nitrosamines and azido compounds in these compounds. Both because we were unable to confirm full duration of ARB therapy and due to the small study population, our study cannot compare this risk of ARB treatment against therapeutic benefits suggested in this work. However, this risk should be accounted for in prospective studies evaluating ARB use in the setting of extant lung cancer.

Several limitations of this work warrant discussion. First, the retrospective design of the study introduces the possibility of unmeasured confounding variables that could contribute to these effects. Indeed, the very same unmeasured factors that led some patients to be prescribed ARBs rather than ACEIs could account for the discrepancy in outcomes. The relatively small sample size, particularly of patients taking ARBs, limit both the reliability and generalizability of our findings. We did not have access to stored samples from our patient population, so neither an association with TGF-β levels nor other biological correlates for these responses could be assessed. Toxicity data were captured from explicit mention radiation oncology visit documentation and radiology reports. Because our institution does not use structured capture of toxicity data, and toxicity documentation is physician-specific, our review likely did not capture all of the grade 1–2 treatment toxicity. Toxicity data were only reviewed up to six months after treatment, so toxicity development outside of that window were not reflected in this paper.

## Conclusions

The present work provides provocative hypothesis generating data suggesting outcomes for patients with early stage NSCLC undergoing SBRT may be improved by the addition of ARB treatment. Importantly, ARBs have a well-studied safety profile, are well-tolerated, and cost-effective, offering the possibility of improving survival while avoiding both medical and financial toxicity. This research should be validated in larger retrospective and prospective studies to demonstrate reproducibility and generalizability. Overall, the wide availability and low cost of these drugs coupled with the findings of increased OS and RFS in early stage lung cancer patients undergoing SBRT could be an important and simple step in improving care for patients with NSCLC.

## Data Availability

The datasets used and/or analysed during the current study are available from the corresponding author on reasonable request
